# *TNF* ^*ΔARE*^ Pigs: A Translational Crohn’s Disease Model

**DOI:** 10.1093/ecco-jcc/jjad034

**Published:** 2023-02-23

**Authors:** Thomas Winogrodzki, Amira Metwaly, Alessandro Grodziecki, Wei Liang, Bernhard Klinger, Tatiana Flisikowska, Konrad Fischer, Krzysztof Flisikowski, Katja Steiger, Dirk Haller, Angelika Schnieke

**Affiliations:** Chair of Livestock Biotechnology, School of Life Sciences, Technical University of Munich, Freising, Germany; Chair of Nutrition and Immunology, School of Life Sciences, Technical University of Munich, Freising, Germany; Chair of Livestock Biotechnology, School of Life Sciences, Technical University of Munich, Freising, Germany; Chair of Livestock Biotechnology, School of Life Sciences, Technical University of Munich, Freising, Germany; Chair of Livestock Biotechnology, School of Life Sciences, Technical University of Munich, Freising, Germany; Chair of Livestock Biotechnology, School of Life Sciences, Technical University of Munich, Freising, Germany; Chair of Livestock Biotechnology, School of Life Sciences, Technical University of Munich, Freising, Germany; Chair of Livestock Biotechnology, School of Life Sciences, Technical University of Munich, Freising, Germany; Comparative Experimental Pathology, Institute of Pathology, Technical University of Munich, Munich, Germany; Chair of Nutrition and Immunology, School of Life Sciences, Technical University of Munich, Freising, Germany; Chair of Livestock Biotechnology, School of Life Sciences, Technical University of Munich, Freising, Germany

**Keywords:** Inflammatory bowel disease [IBD], Crohn’s disease, pig/swine model

## Abstract

**Background and Aims:**

Crohn’s disease [CD] is a major subtype of inflammatory bowel diseases [IBD] with increasing incidence and prevalence. Results of studies using available small and large animal models are often poorly translatable to patients, and few CD models show small intestinal pathology. Due to its similarities to humans, the pig has emerged as a highly suitable translational disease model, particularly for testing novel nutritional and technological interventions. Our goal was to develop a physiologically relevant porcine CD model to facilitate translation of findings and interventions towards the clinic.

**Methods:**

We generated pigs bearing a 93-bp deletion of the adenosine–uracil-rich element [ARE] and a constitutive-decay element within the 3ʹ untranslated region of the *TNF* gene. Comparative analysis of physiological, molecular, histological and microbial characteristics was performed between wild-type, *TNF*^*ΔARE/+*^ and *TNF*^*ΔARE/ΔARE*^ animals. Alterations in the microbiome were compared to the *TNF*^*ΔARE*^ mouse model and IBD patients.

**Results:**

*TNF* ^*ΔARE*^ pigs recapitulate major characteristics of human CD, including ulcerative transmural ileocolitis, increased abundance of proinflammatory cytokines, immune cell infiltration and dysbiotic microbial communities. 16S rRNA gene amplicon sequencing revealed enrichment in members belonging to *Megasphaera*, *Campylobacter*, *Desulfovibrio*, *Alistipes* and *Lachnoclostridum* in faecal or mucosa-associated bacteria compared to wild-type littermates. Principal components analysis clustering with a subset of *TNF*^*ΔARE/+*^ mice and human IBD patients suggests microbial similarity based on disease severity.

**Conclusions:**

We demonstrate that the *TNF*^*ΔARE*^ pig resembles a CD-like ileocolitis pathophenotype recapitulating human disease. The ability to conduct long-term studies and test novel surgical procedures and dietary interventions in a physiologically relevant model will benefit future translational IBD research studies.

## 1. Introduction

Crohn’s disease [CD] is one of the two main subtypes of inflammatory bowel diseases [IBD] characterized by patchy transmural inflammation in the entire digestive tract, predominantly affecting the terminal ileum and proximal colon.^1^ CD is a multifactorial disease driven by complex gene–environment interactions following patterns of industrialization.^2^ Genome-wide association studies have identified a variety of genetic risk alleles pointing towards a disruption of microbe–host interactions.^3^ The underlying molecular mechanisms of IBD and specific microbial and metabolic signatures have been elucidated using spontaneous, chemically induced and genetically engineered mouse or large animal models.^4–6^ However, CD-like inflammation with manifestation of small intestinal disease rarely occurs in currently available animal models of IBD, with mouse models being the most widely applied [e.g. SAMP/YitFc, *Xiap*^−/−^, *Xbp1*^−/−^, *TNF*^*ΔARE*^]. Yet, due to their short lifespan and size, mouse models are of limited use for the assessment of human-scale technologies and methods and in some cases are less suitable as disease models compared to species that are genetically, physiologically and anatomically more closely related to humans.^7,8^

Our goal was therefore to generate a physiologically relevant animal model that reflects the human disease phenotype of CD as accurately as possible. It should be able to predict the influence of altered nutrition or microbiome on human or animal health and should represent a reliable preclinical model to assess new therapeutic approaches. Because of their comparatively large size and lifespan, large animal models are particularly well suited for testing technologies and methods developed for human clinical use, as well as for long-term studies and the collection of multiple fluid or tissue biopsies from a single animal, consistent with the 3Rs rules—replacement, reduction, refinement.^9^ In the past, in particular non-human primates [NHPs] and dogs have been used as large animal models for preclinical research because they spontaneously develop IBD.^5,6^ However, animal studies with NHPs, despite their obvious similarities to humans, are ethically controversial, costly and carry the risk of transmitting zoonotic diseases.^10^ Canines are highly sensitive to intestinal disease and, therefore, often suffer from high mortality rates, mainly due to intestinal ischaemia. This is in addition to society’s growing rejection of animal testing on dogs.^10^ On the other hand, the pig has emerged as an exceptionally well-suited large animal model for intestinal diseases because of its strong physiological, anatomical, genomic, immunological and nutritional similarities to humans. Porcine models have long been used to study the effect of human nutrition on metabolic syndromes, obesity and food allergies.^11^ Both pig^12^ and human bacterial libraries^13^ are available to elucidate the effect of specific bacterial strains on IBD. Pigs can be fed a human diet and faecal microbiota transfer from humans to pigs results in a gut microbiota closely resembling that of the human donor.^14,15^ A recent study of a large pig population assessed the effect of host genotype on the composition of the intestinal microbiota, demonstrating the high value and applicability of digestive disease research.^16^ Being also used for food production the opposition towards porcine models, especially in areas of great clinical need, is considerably lower compared to dogs or NHPs.

Here we report the generation and characterization of a porcine model for CD carrying a 93-bp deletion in the 3ʹ-untranslated region [UTR] of *TNF* [*TNFα*], which deleted the transcript-destabilizing AU-rich element [ARE] and a constitutive decay element [CDE] similar to the *TNF*^*ΔARE*^ mouse model.

## 2. Materials and Methods

### 2.1. Ethics statement

Animal experiments were approved by the Committee on Animal Health and Care of the local government body of the state of Upper Bavaria [ROB 55.2-2532.Vet_02-18-56, 55.2-1-54- 2531-99-13] and performed according to the German Animal Welfare Act and European Union Normative for Care and Use of Experimental Animals.

### 2.2. Generation of *TNF*^*ΔARE*^ pigs

For CRISPR/Cas9-mediated excision of the 93-bp fragment containing the ARE and CDE1 element, two single guide RNAs [sgRNAs] were designed using CRISPOR.^17^ Both U6-gRNA-scaffold sequences were cloned into px330-U6-Chimeric_BB-CBh-hSpCas9 plasmid2 [Addgene no. 42230] [pX330-2gRNAs]. The pX330-2gRNAs plasmid DNA was microinjected into *in vitro* fertilized porcine oocytes followed by laparoscopic embryo transfer, as previously described.^18,19^ Genomic DNA was isolated from ear biopsies using a GenElute Mammalian Genomic DNA Kit [Sigma]. Detection of the 93-bp deletion was determined by PCR using GoTaq polymerase [Promega] and primer pairs: Fwd: 5ʹ-GGGTTTGGATTCCTGGATGC-3ʹ, Rev: 5ʹ-GCGGTTACAGACACAACTCC-3ʹ. Thermal cycling parameters were: 95°C, 2 min; [35×] 95°C, 45 s, 60°C, 45 s, 72°C, 30 s; 72°C, 5 min. Amplicons were verified by Sanger sequencing [Eurofins Genomics]. sgRNA off-target sites were predicted using CRISPOR. Five highest scoring potential off-targets were analysed by PCR and Sanger sequencing.

### 2.3. Macrophage culture and RNA half-life analysis

EDTA-blood was collected from anaesthetized pigs. Peripheral blood mononuclear cells [PBMCs] were isolated by Ficoll-density gradient centrifugation. PBMCs were cultured in 10 mL RPMI 1640, 10% fetal calf serum [FCS], 1% GlutaMax [Sigma], 1% Pen-Strep/Amphotericin B and 10^4^ U/mL recombinant poGM-CSF [Biotechne] for 7 days. Macrophages were divided into four groups: [1] no supplements, [2] supplemented with 0.1 µg/mL lipopolysaccharide [LPS; InvivoGen], [3] with LPS and 150 µg/mL Polymyxin B [Invivogen] and [4] with LPS and after 45 min with 10 µg/mL Actinomycin D [Calbiochem]. Macrophages were harvested at 0, 45 and 90 min and frozen at −80°C until processing.

### 2.4. RNA isolation and quantification

RNA was isolated from gut mucosal biopsies using a Monarch Total RNA Miniprep Kit [NEB] according to the manufacturer’s protocols. cDNA was generated using LunaScript RT Master Mix Kit [NEB] according to the manufacturer’s protocol. Real-time quantitative PCR [qPCR] was performed using the Fast SYBR Green Master Mix [Sigma] in a QuantStudio 5 Real-Time-PCR-Cycler [Thermofisher Scientific] and the following primer pairs: TNF, Fwd: 5ʹ-GGGCTTATCTGAGGTTTGAG-3ʹ, Rev: 5ʹ-TTCTGCCTACTGCACTTCGA-3ʹ; IL6, Fwd: 5ʹ-TCTGCAATGAGAAAGGAGATGTG-3ʹ, Rev: 5ʹ-AGGTTCAGGTTGTTTTCTGCC-3ʹ; IL8, Fwd: 5ʹ-CTGTGAGGCTGCAGTTCTG-3ʹ, Rev: 5ʹ-GTGATTGAGAGTGGACCCCA-3ʹ. For transcript normalization, housekeeping genes GAPDH [Fwd: 5ʹ-TTCCACGGCACAGTCAAGGC-3ʹ, Rev: 5ʹ-GCGGTTACAGACACAACTCC-3ʹ], β-actin [Fwd: 5ʹ-TCCCTGGAGAAGAGCTACGA-3ʹ, Rev: 5ʹ-GCAGGTCAGGTCCACAAC-3ʹ] and RPS28 [Fwd: 5ʹ-GTTACCAAGGTTCTGGGCAG-3ʹ, Rev: 5ʹ- CAGATATCCAGGACCCAGCC-3ʹ] were selected based on NormFinder and BestKeeper.^20,21^ One-way ANOVA was performed, followed by Tukey’s test for statistical evaluation using GraphPad Prism 8.

### 2.5. Protein quantification via Western blot

Ileal and colonic proteins were obtained by tissue homogenization in NP-40 buffer with 1× cOmplete Mini Protease Inhibitor Cocktail [Roche]. Protein concentrations were determined using the Bradford assay. Protein lysates were separated by semi-dry Western blot (anti-TNF, 1:1000, Invitrogen [14-7321-85]; anti-GAPDH, 1:3000, Sigma [G8795]; anti-ZO1, 1:250, ThermoScientific [61-7300]; anti-occludin, 1:666, LSBio [LS-B5737]). Blots were developed using Pierce ECL Plus Western Blotting Substrate [ThermoScientific].

### 2.6. Protein quantification via ELISA

Faecal calprotectin content was measured by using faecal water and a pig calprotectin ELISA Kit [Cusabio, CSB-EQ013485PI]. Faecal water was isolated by diluting 50 mg of faeces in 400 µL PBS followed by thorough vortexing. Homogenates were centrifuged at 500 *g* for 5 min, and the supernatant was collected for a second round of centrifugation at 6000 *g* for 5 min. The supernatant was 5-fold diluted in 1× assay reagent for protein quantification.

### 2.7. Histology and immunohistochemistry

Macroscopic findings during necropsy were documented, and images were reviewed by a board-certified veterinary pathologist. Representative specimens for histology were collected from rectum, proximal colon, caecum, distal ileum, proximal jejunum and proximal duodenum, fixed in 10% neutral-buffered formalin and embedded in paraffin [FFPE]. H&E-stained tissue sections [2 µm] were assessed for histological changes. Immunohistochemical stainings were performed on 3.5-μm FFPE tissue sections as described previously^22^ using the following antibodies: anti-Ki67, 1:300, Invitrogen [MA5-14520]; anti-CD3, 1:100, Southern Biotech [4511-01]; and anti-IBA1, 1:2000, Wako FujiFilm [019-19741]. Periodic Acid-Schiff-Alcian blue [Pas/AB] staining was performed as previously described.^22^ Quantification of lamina propria CD3 [T-cells] and IBA1 [macrophages] was performed on randomly selected areas and the ratio of positive/negative cells was calculated. Pas/AB^+^ and crypt Ki67^+^ cells were quantified by counting positive cells per crypt area. Statistical evaluation was performed as described above. Immunofluorescence staining was performed on 3.5-µm FFPE colonic sections using the following antibodies: anti-ZO1, 1:50, ThermoScientific [61-7300]; anti-occludin, 1:100, LSBio [LS-B5737]; and goat-anti-rabbit-Alexa Fluor Plus 594, 1:100, Invitrogen [A11012]. Confocal laser scanning microscopy was performed with an Olympus FV 3000 confocal microscope with a 60×/NA1.2/WD0.28 UPLSAPO60XS2 objective and water immersion lens, Olympus four-channel TruSpectral detection system and the Olympus FV 3000 Imaging Software, Cells Desktop Version 1 of the Center for Advanced Light Microscopy [CALM, Technical University of Munich, Germany].

### 2.8. Porcine samples for microbiota profiling

Wild-type and *TNF*^*ΔARE*^ littermate pigs were co-housed at the Technical University of Munich. No anti-diarrhoeal medication was administered. Piglets were raised with their mothers until weaning. All pigs received vaccinations against *Lawsonia intracellularis*, pneumococcal disease [pneumococcal polysaccharide vaccine, PPV], porcine reproductive and respiratory syndrome [PRRS], influenza, mycoplasma and porcine circovirus type 2 [PCV2]. All animals received the same diet [HEMO U 134 pellets; LikraWest] and water *ad libitum*. Faeces were collected and consistency was assessed monthly starting shortly after birth by digital sampling. Gut luminal and mucosal tissue samples were collected together with control samples of ambient air during necropsy and stored in Lysing Matrix B tubes [MP Biomedicals] filled with 500 µL Stool DNA Stabilizer [Invitek] at −80°C for downstream analysis.

### 2.9. Murine samples for microbiota profiling

*TNF* ^*ΔARE*^ mice and wild-type littermates were imported from Case Western Reserve University, Cleveland, and housed under germ-free [GF] conditions. To establish a specific-pathogen-free [SPF] microbiota-colonized colony, 8-week-old GF mice were colonized with SPF-derived microbiota and were maintained under SPF housing conditions for three generations. Faecal samples were collected over time [8, 10, 12 and 18 weeks] and frozen at −20°C for downstream analysis.

### 2.10. Human samples for microbiota profiling

A total of 133 faecal samples from 29 CD patients with up to 5-year follow-up following haematopoetic stem cell transplantion [HSCT] were included in the analysis. Fresh faecal samples were collected either at the clinic or at home by the patients using a stool collection kit within 24 h prior to the study visit. Patients were instructed to keep the samples stored in the home freezer until transported to the study site, as described previously.^23^ Clinical assessments with measurement of the Crohn’s disease activity index [CDAI] and biomarkers including C-reactive protein and faecal calprotectin were performed at baseline [before HSCT]. Metagenomic DNA extraction and 16S rRNA gene sequencing profiling were performed as described previously.^23^

### 2.11. High-throughput 16S rRNA gene amplicon sequencing

Metagenomic DNA was extracted as previously described.^24^ Briefly, cells were mechanically lysed in DNA stabilization buffer and extracted using phenol/chloroform/isoamyl alcohol [25:24:1, by vol.]. Following heat treatment of cells and centrifugation, supernatants were treated with RNase. DNA was purified with a NucleoSpin gDNA Clean-up Kit [Macherey-Nagel], following the manufacturer’s instructions. Amplification with primers 341F-ovh and 785r-ovh and sequencing of the V3/V4 region of 16S rRNA genes was performed as previously described.^25,26^ In total, 227 porcine and 98 mouse samples were sequenced in paired-end mode [PE275] using a MiSeq system [Illumina] according to the manufacturer’s instructions.

### 2.12. Amplicon sequence analysis

Raw 16S rRNA amplicon reads were pre-processed using the Integrated Microbial Next Generation Sequencing pipeline.^27^ Five nucleotides on the 5ʹ end and 3ʹ end were trimmed for the R1 and R2 read [trim score 5] and an expected error rate of 1. Detected chimeric sequences were removed using UCHIME.^28^ Sequences with relative abundance <0.25% and <300 and >600 nucleotides were excluded from analysis. A zero-radius operational taxonomic unit [zOTU] table was constructed considering all reads before quality filtering. Downstream analysis was performed using Rhea.^29^ Taxonomy assignment was done using RDP classifier version 2.11 and confirmed using the SILVA database.^30^ For phylogenetic analyses, maximum-likelihood trees were generated by FastTree based on MUSCLE alignments in MegaX.^31^ Alpha-diversity analysis was computed using community richness and Shannon’s effective number of species. Beta-diversity analysis was performed using generalized UniFrac distances. Permutational multivariate analysis of variance [PERMANOVA] was performed for statistical evaluation of beta-diversity.

## 3. Results

### 3.1. Generation of *TNF*^*ΔARE*^ pigs

Binding motifs of RNA-degrading factors in the 3ʹ-UTR of the *TNF* gene were identified by sequence alignments with human and murine orthologues. The tumour necrosis factor [TNF] class II AREs, which possess at least two overlapping UUAUUUA[U/A][U/A] nonamers,^32^ were located at position 445–516 bp downstream of the TNF stop codon. The CDEs were detected at positions 501–518 bp [CDE1] and 575–589 bp [CDE2] downstream of the stop codon.

**Figure 1.**
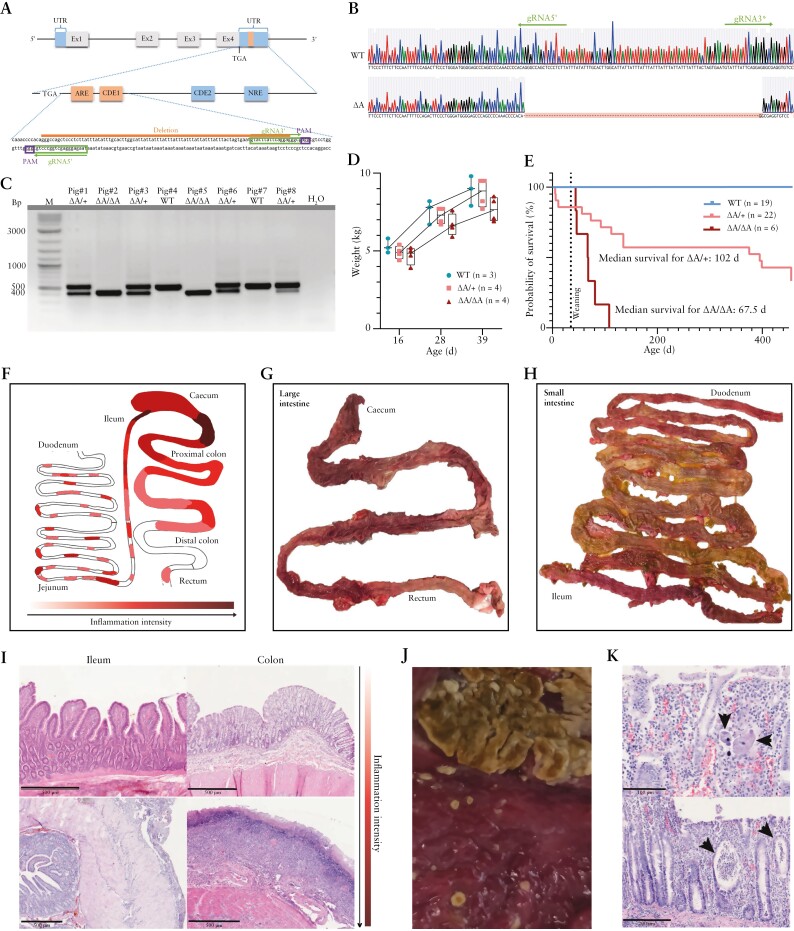
[A] Genome editing strategy for the deletion of the ARE and CDE1 elements. Expanded view shows the 3ʹ-untranslated region [UTR] and excised sequence with the gRNA binding sites [marked in green]. ARE, CDE and NRE elements are shown as boxes, and the excised fragment [93 bp] is marked in orange. [B] Sanger sequencing showing expected excision of 93 bp. [C] Genotyping PCR of genome-edited founder piglets [pigs 1–8 from one of two litters born]. Expected size for the amplified wild-type TNF fragment of 524 bp, and for *TNF*^*ΔARE*^ of 431 bp. M, size marker. [D] Weight gain of wild-type, hetero- and homozygous *TNF*^*ΔARE*^ sibling pigs aged 16–39 days. [E] Kaplan–Meier curve indicating the time point at which animals were killed due to deteriorating health. The median survival times for euthanized *TNF*^*ΔARE/+*^ and *TNF*^*ΔARE/ΔARE*^ pigs are given. [F] Schematic representation of intestinal tract and sites of inflammation. Darker red colouring indicates sites of increased inflammation. [G, H] Representative images of inflamed large and small bowel. Macroscopic signs of inflammation were continuous throughout the large intestine, and segmental in the small intestine. [I] Representative images of H&E-stained colonic and ileal gut sections. Shown are mildly inflamed [top] and highly inflamed [bottom] tissue sections with crust formation [colon] and serositis [ileum]. Scale bars = 500 µm. [J] Diphtheritic membranes in the caecum of a *TNF*^*ΔARE/ΔARE*^ pig. [K] Histopathological findings of invasive *Balantidium coli* [top] and crypt abscesses [bottom]. Scale bars = 100 µm [top] and 200 µm [bottom].

Excision of the ARE and CDE1 sequences was performed by microinjection of a Cas9 expression vector, which also encodes the two gRNAs, into *in vitro* generated porcine zygotes [Figure 1A]. Five embryo transfers were carried out, resulting in two pregnancies and the birth of ten piglets of which two showed biallelic excision of the *ARE/CDE1* sequence [*TNF*^*ΔARE/ΔARE*^, pigs 2 and 5], and five a monoallelic deletion [*TNF*^*ΔARE/+*^, pigs 1, 3, 6, 8 and 9] [Figure 1B and C]. Two of the latter piglets [nos. 8, 9] showed in addition mosaicism and/or InDel mutations on the second allele, which was eliminated from the line after breeding with wild-type animals. The founder animals [nos. 6, 8, 9] used for breeding were screened for the five most likely off-targets and none were detected. Breeding resulted in 11 *TNF*^*ΔARE/+*^ F1 and ten F2 offspring [seven *TNF*^*ΔARE/+*^, three *TNF*^*ΔARE/ΔARE*^]. A total of 13 male and ten female *TNF*^*ΔARE/+*^, and two male and three female *TNF*^*ΔARE/ΔARE*^ piglets were born. All progeny carried the identical sequence of the mutant allele.

### 3.2. *TNF*^*ΔARE*^ pigs show clinical manifestations and inflammatory alterations of the intestine

After weaning, ~50% of *TNF*^*ΔARE/+*^ animals showed reduced weight gain, and increased Bristol stool score that positively correlated with faecal calprotectin levels [Pearson’s *r*: 0.5242; *p*_adjusted_: 0.51]. This phenotype was more pronounced in *TNF*^*ΔARE/ΔARE*^ pigs [Figure 1D; Supplementary Figure S1A and B]. Affected heterozygous and homozygous pigs were killed when termination criteria [chronic diarrhoea, severe weight loss, apathy] manifested. Median lifespan was 102 days for heterozygous *TNF*^*ΔARE/+*^ pigs and 67.5 days for homozygous *TNF*^*ΔARE/ΔARE*^ pigs [Figure 1E]. Macroscopically, *TNF*^*ΔARE*^ animals had a more fragile intestinal wall when sampled and showed intestinal oedema and haemorrhage throughout the colon, segmental in the small intestine, frequently including the caecum [Figure 1F–H]. As with mice, inflammation intensity varied between pigs of the same genotype [Figure 1I]. In *TNF*^*ΔARE/ΔARE*^ pigs the most severe alteration was ulcerative enteritis, sometimes covered with diphtheritic membranes in the caecum [Figure 1J]. Microscopically, 67% [4/6] of pathologically evaluated *TNF*^*ΔARE/+*^ animals showed ileitis and/or colitis with mixed infiltrations of the lamina propria often extending into the tela submucosa accompanied by fibrino-suppurative serositis [Figure 1I]. In rare cases, extensive crypt abscesses [Figure 1K] and frequent herniation of crypts throughout the lamina muscularis mucosae or ulceration of the lamina propria mucosae with mixed infiltration and development of fibroangioblastic granulation tissue were observed. As expected from the macroscopic analysis, *TNF*^*ΔARE/ΔARE*^ pigs showed a higher degree of intestinal abnormalities, including strong ulcerative inflammation with crust formation. In one case, an invasion of *Balantidium coli* into the mucosa was observed [Figure 1K].

**Figure 2.**
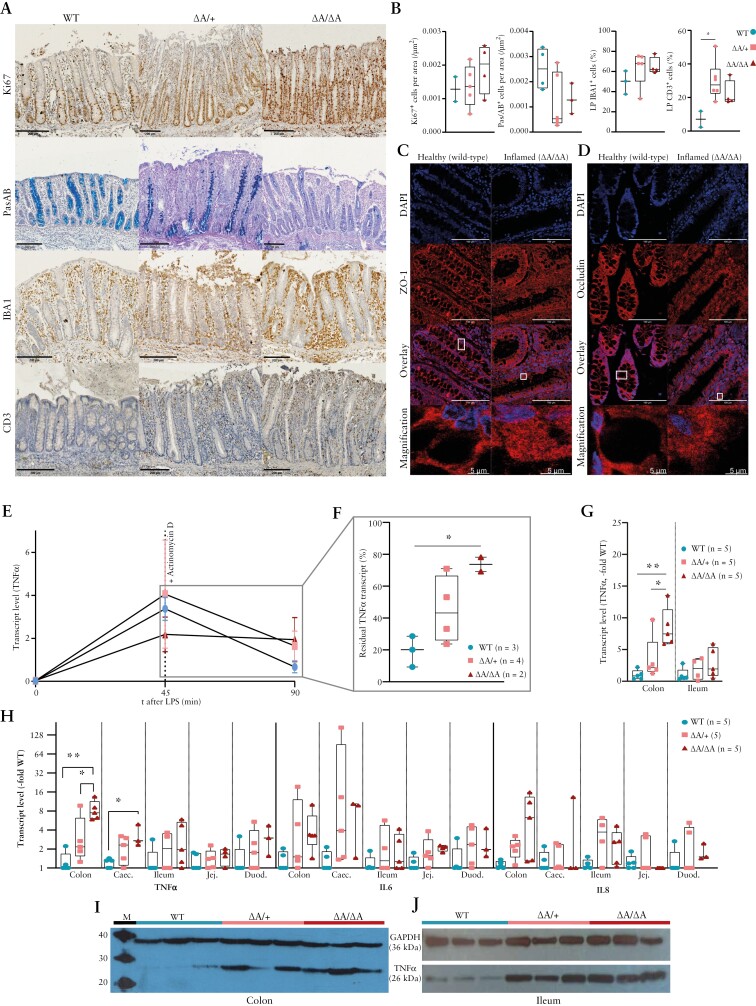
[A, B] Immunohistology and statistical evaluation of colon samples from wild-type and *TNF*^*ΔARE*^ pigs showing an increase in Ki67^+^, CD3^+^ and IBA1^+^ cells and decrease in Pas/AB^+^ cells. Scale bars = 200 µm. [C, D] Confocal laser immunofluorescence scanning microscopy images of colonic sections stained for DAPI and ZO-1 or Occludin, respectively. Scale bars = 100 µm. Representative single cells were selected and shown magnified. Original signal intensities were adjusted for clear visualization of defined or diffuse antigen localization. [E, F] *TNF* mRNA levels and mRNA half-life assessed in macrophages from wild-type and *TNF*^*ΔARE*^ pigs. [G] Comparison of *TNF* transcript levels in colonic and ileal mucosal biopsy samples shown as multiples of the average wild-type value. [H] Comparison of *TNF*, *IL-6* and *IL-8* mRNA levels in intestinal samples shown as multiples of the average wild-type value on a log_2_ scale. Caec, caecum; Duod, duodenum; Jej, jejunum. [I, J] TNF and GAPDH protein expression in colonic [K] and ileal samples [L] from three wild-type, three *TNF*^*ΔARE/+*^ and three *TNF*^*ΔARE/ΔARE*^ pigs. M, size marker.

Immunohistochemistry confirmed increased cell proliferation [Ki67^+^], increased leukocyte and lymphocyte infiltrations [IBA1^+^, CD3^+^], and decreased number of mucus-secreting goblet cells [Pas/AB^+^] in inflamed areas, especially in the ileocolonic region [Figure 2A and B]. Confocal laser immunofluorescence microscopy revealed a diffuse re-localization of tight junction proteins ZO-1 and occludin in inflamed tissue [Figure 2C and D], and reduced protein levels for ZO-1 and occludin in the proximal colon of most *TNF*^*ΔARE*^ mutants compared to wild-type pigs [Supplementary Figure S1B–D]. Complete blood counts revealed an increased monocyte count without significant changes in total white blood cells, basophilia, increased urea/creatinine ratio, hypoalbuminaemia and elevated serum Cu/Zn [Supplementary Figure S1F]. No signs of rheumatoid arthritis were observed.

### 3.3. Deletion of ARE/CDE1 results in increased *TNF*-mRNA half-life, transcript abundancy and protein levels

*TNF* is expressed by many different cell types, with highest expression in cells of the monocytic lineage, such as macrophages.^33^ Therefore, macrophage cultures were established from PBMCs to investigate if the *TNF* mutation affects mRNA half-life. Transcription of *TNF* in macrophages was stimulated with LPS and inhibited after 45 min by the addition of actinomycin D. Prior to stimulation, *TNF* mRNA levels were slightly higher in macrophages isolated from *TNF*^*ΔARE/ΔARE*^ and *TNF*^*ΔARE/+*^ pigs compared to controls [2- and ~1.2-fold]. LPS stimulation resulted in an increase in *TNF* transcript levels at 45 min compared with resting macrophages in all genotypes. Inhibition of transcription through addition of actinomycin D resulted in a decrease of *TNF* mRNA levels. After 45 min of incubation with actinomycin D, the detected transcript levels were ~0.19% [wild-type], ~0.45% [*TNF*^*ΔARE/+*^] and ~0.74% [*TNF*^*ΔARE/ΔARE*^] compared to levels before the addition of this transcription inhibitor [Figure 2F and G]. Thus, the calculated half-lives of *TNF* mRNA in macrophages were ~19 min for wild-type, ~58 min for heterozygous and ~708 min for homozygous *TNF*^*ΔARE*^ pig samples. Consistent with these results, colonic *TNF* mRNA expression was 7.4-fold higher [*p*_adj_ = 0.0027] in *TNF*^*ΔARE/ΔARE*^ pigs and 2.2-fold [not significant] higher in *TNF*^*ΔARE/+*^ swine compared to wild-type pigs [Figure 2H and I]. The elevated *TNF* mRNA expression resulted in increased TNF protein level in the colon and ileum of *TNF*^*ΔARE*^ pigs, as shown by Western blot [Figure 2J and K].

Next, the effect of the increased *TNF* expression on its downstream targets was assessed. In colon tissue from *TNF*^*ΔARE*^ pigs, mRNA expression of the key pro-inflammatory cytokine interleukin-6 [IL-6]^34^ was 3.4-fold increased and the potent neutrophil chemoattractant IL-8^35^ was 6.3-fold increased compared to wild-type controls. The increase in transcript abundance of all three target genes was observed throughout the gut, but most markedly in the ileocolonic region [Figure 2I].

### 3.4. Intestinal inflammation is linked to luminal and mucosa-associated bacterial dysbiosis in *TNF*^*ΔARE*^ pigs

The relevance of dysbiotic microbial communities in initiating CD-like inflammation in *TNF*^*ΔARE*^ mice was previously shown.^36^ To characterize the changes in intestinal microbiota composition in relation to inflammation severity in *TNF*^*ΔARE*^ pigs, we performed 16S rRNA gene sequencing on 166 mucosal tissue biopsies and 61 stool samples from seven wild-type, ten *TNF*^*ΔARE/+*^ and six *TNF*^*ΔARE/ΔARE*^ pigs. Samples were obtained from defined positions along the gastrointestinal tract. The microbial signatures were then compared with luminal and mucosal samples obtained from *TNF*^*ΔARE/+*^ mice and a previously published cohort of human IBD patients.^23^

**Figure 3.**
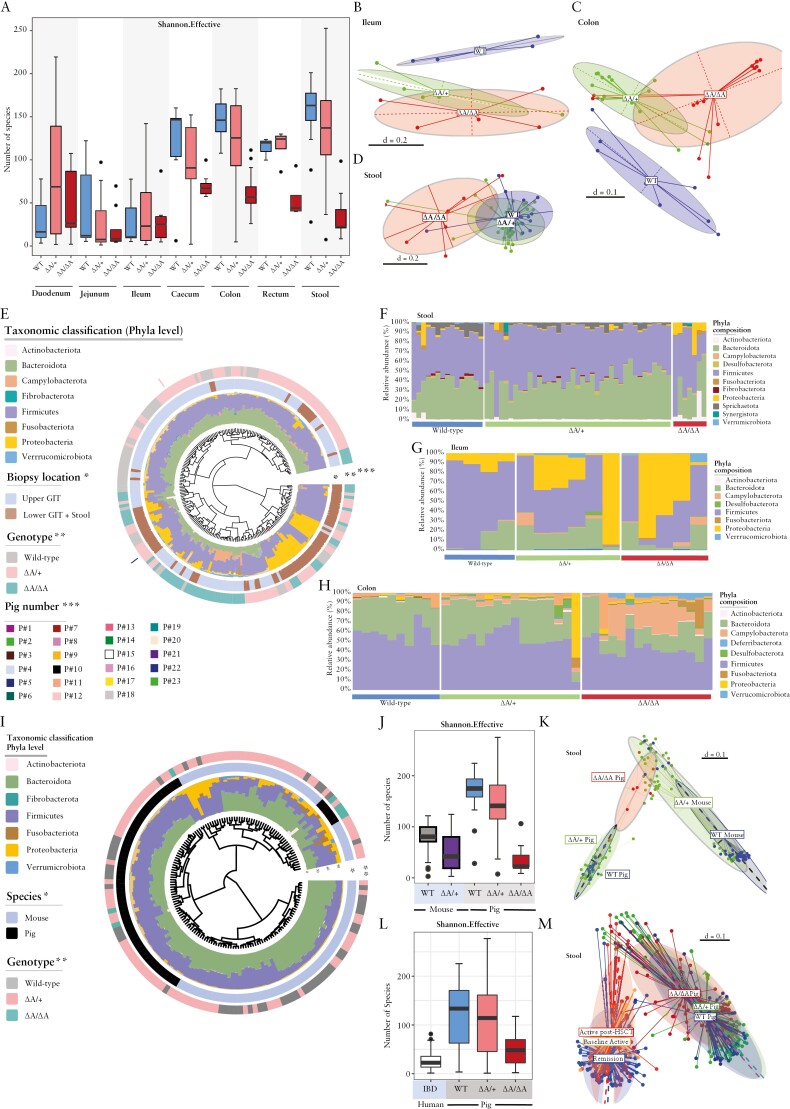
[A] Alpha-diversity of the luminal and mucosa-associated microbiota. Shannon an effective number of species. Sample locations and genotypes are indicated. [B–D] Multidimensional scaling [MDS] plots of microbial profiles of faecal, ileal and colonic samples stratified by genotype, respectively. [E] Phylogenetic tree showing the similarities between microbiota profiles based on generalized UniFrac distances in luminal [*n* = 61] and mucosa-associated microbiota [*n* = 166] derived from 23 pigs [seven wild-type, 16 *TNF*^*ΔARE*^]. Individual taxonomic composition at the phylum level is shown as stacked bar plots around the phylogram. Innermost ring shows stratification based on sample type, upper GI tract samples [blue] and lower GI tract and stool samples [brown] and denoted by an asterisk [*]; the second ring shows stratification based on genotype, wild-type [grey], *TNF*^*ΔARE/+*^ [pink] and *TNF*^*ΔARE/ΔARE*^ [green] and denoted by two asterisks [**]. Bars in the outer ring of the figure indicate samples derived from each pig and denoted by three asterisks [***]. [F–H] Taxonomic composition at the phylum level in faecal, ileal and colonic samples stratified by genotype. [I] Phylogenetic tree showing the similarities between microbiota profiles based on generalized UniFrac distances in luminal microbiota derived from wild-type and *TNF*^*ΔARE*^ pigs and mice. Individual taxonomic composition at the phylum level is shown as stacked bar plots around the phylogram. Innermost ring shows stratification based on species, mouse or pig; and the outer ring shows stratification based on genotype, wild-type [grey], *TNF*^*ΔARE/+*^ [pink] and *TNF*^*ΔARE/ΔARE*^ [green]. [J] Alpha-diversity of luminal microbiota from wild-type and *TNF*^*ΔARE*^ pigs and mice is shown as Shannon effective number of species. [K] MDS plot of microbial profiles of faecal samples stratified by species [mouse or pig] and genotype, respectively. [L] Alpha-diversity of luminal and mucosal microbiota from human IBD patients and wild-type and T*NF*^*ΔARE*^ pigs is shown as Shannon effective number of species. [M] MDS plot of microbial profiles of samples stratified by species [human or pig] and disease activity or genotype, respectively.

Alpha-diversity was reduced in the large intestine and jejunum of *TNF*^*ΔARE*^ pigs, while it was enriched in duodenum and ileum [Figure 3A]. Beta-diversity analysis showed separation of microbial profiles in ileal and colonic mucosa of wild-type, *TNF*^*ΔARE/+*^ or *TNF*^*ΔARE/ΔARE*^ pigs, which was weakly reflected in stool [Figure 3B–D]. Comparing individual microbiota compositions confirmed diverse ecosystems dominated by the two major phyla Firmicutes and Bacteroidetes in mucosal biopsies from duodenum, jejunum and ileum (upper gastrointestinal [GI] tract) in contrast to faecal microbiota or colon and cecum mucosal biopsies [lower GI tract] which were dominated by Firmicutes and Proteobacteria [Figure 3E, innermost ring and bar plots]. Microbiota profiling showed clear clustering in terms of inflammation severity in the different genotypes [Figure 3E, middle ring]. Longitudinal microbial profiling of luminal and mucosa-associated microbiota showed individual-specific clustering of microbiota composition [Figure 3E, outermost ring]. Taxonomic classification at the phylum level showed significantly increased relative abundance of the phylum Proteobacteria in faecal and ileal-associated bacterial communities of *TNF*^*ΔARE*^ pigs compared to wild-type littermates [Figure 3F And G]. On the other hand, an enrichment of Fusobacteriota and Campylobacterota was observed in colon-associated microbial communities of *TNF*^*ΔARE*^ pigs [Figure 3H]. Linear discriminant analysis effect size [LEfSe] analysis showed an enrichment in members of the genera *Helicobacter*, *Megasphaera*, *Campylobacter*, *Desulfovibrio*, *Alistipes* and *Lachnoclostridum* among others in faecal or mucosa-associated bacteria of *TNF*^*ΔARE*^ pigs compared to wild-type littermates [Supplementary Figure S2A–C].

Calprotectin protein concentrations were measured in faeces from highly and mildly inflamed pigs for two consecutive post-weaning time points t1 and t2 to examine whether changes in faecal bacterial composition precede inflammation. The stool samples were classified as severely [‘High’] or mildly [‘Low’] inflamed, based on the calprotectin concentration with a threshold of 30 ng/mL [Supplementary Figure S2D]. At t1, 2/7 [29%] and at t2, 4/7 [57%] specimens had a calprotectin concentration above 30 ng/mL in the stool samples [Figure S2D]. Overall, the average concentration of calprotectin was 1.6-fold [n.s.] higher at t2 compared to t1. β-Diversity analysis revealed weak inflammation-dependent shifts of microbiota profiles [Supplementary Figure S2E]. Calprotectin levels at t2 had a weak impact on the microbiota profiles of the respective samples at t1. Samples that were highly inflamed at t2 [‘HI’] more closely clustered together with the respective samples at t1 when compared to samples that were mildly inflamed at t2 [‘MI’], suggesting that a change in the gut microbiome precedes the inflammation detectable in the stool [Supplementary Figure S2F].

The comparison of luminal microbiota between *TNF*^*ΔARE*^ pigs and mice showed clear separation of microbiota profiles with a subset of *TNF*^*ΔARE/ΔARE*^ pigs clustering with *TNF*^*ΔARE/+*^ mice, suggesting a species-independent microbial signature of intestinal inflammation. As expected, wild-type pigs showed significantly higher bacterial community richness and diversity compared to mice, while *TNF*^*ΔARE/ΔARE*^ pigs showed the lowest number of species [Figure 3I–K]. LEfSe analysis identified a set of bacterial genera that are significantly differential between *TNF*^*ΔARE/+*^ pigs and mice. While in mice an increased abundance of *Alistipes*, *Bacteriodes*, *Escherichia coli*, *Fusobacterium* was observed, the *TNF*^*ΔARE/+*^ pigs showed more *Prevotella*, *Lactobacillus*, *Treponema* and *Megasphera*, among others [Supplementary Figure S2G]. Similar results were observed when comparing *TNF*^*ΔARE*^ pigs and the human IBD cohort. We found clear species-specific clustering of microbiota profiles [Supplementary Figure S2H] and a higher bacterial diversity in porcine samples [Figure 3L]. Notably, samples derived from IBD patients with active disease clustered with a subset of *TNF*^*ΔARE/ΔARE*^ and *TNF*^*ΔARE/+*^ pigs [Figure 3M], supporting the assumption of a species-independent microbial signature of IBD.

## 4. Discussion

Despite decades of basic and clinical research, IBD remains a global health problem with rising incidence and prevalence.^2^ The mouse models for CD-like chronic inflammation are ideally suited to basic research, to elucidate the molecular basis of the disease and to assess the interplay between gut epithelium, inflammation and the microbiome. This has undoubtedly contributed to considerable improvements in the treatment of CD.^22,36^ However, major differences between humans and rodents especially with regard to nutrient requirements, the physiology of the GI tract, as well as immunological and metabolic differences hamper the translation of findings into the clinic.^7,37–40^ There is a need for more physiologically relevant translational models. Both humans and pigs are omnivorous and share similarities related to anatomical features of the GI tract. Pigs are a widely accepted model for nutrition research, intestinal microbiota and gut barrier function.^11^ All of this makes them an ideal model for IBD.

*TNF* ^*ΔARE*^ pigs show symptoms characteristic of IBD, including chronic diarrhoea and weight loss, aberrant intestinal morphology and changes in intestinal epithelial cell composition.^1^ Similar to human CD patients, transmural skip lesions within the intestinal tract with ileocolitic predominance, reduced numbers of PAS/AB^+^ cells, increased cell proliferation and increased immune cell infiltrations in inflamed areas were observed.^1^ The blood tests were indicative of intestinal inflammation, malnutrition, gastrointestinal haemorrhage and active disease.^41–43^ Together with the observed diffuse patterning and lower concentration of tight junction proteins in most *TNF*^*ΔARE*^ pigs, fibrinous exudates and gut-penetrating *Balantidium coli*, our findings suggest a disruption of the intestinal barrier with loss of mucosal tolerance. A disrupted intestinal barrier not only facilitates microbial invasion into intestinal tissues but also allows increased passage of oxygen into the intestinal lumen with devastating effects on the intestinal microbiota.^44,45^ In line with these data, we observed a reduction in the often obligate anaerobic genera of Bacteroidota and Firmicutes and an increase in the largely facultatively anaerobic Proteobacteria with a concomitant decrease in bacterial diversity in the ileum of *TNF*^*ΔARE*^ pigs, findings previously reported for IBD patients.^44,46^ Furthermore, an increase of Campylobacterota along with a decline of Bacteroidota in the colon was previously associated with an increased risk of IBD.^47,48^ Compositional changes in the stool microbiota were found to be weakly influenced by future calprotectin levels, indicating that changes in the faecal microbiota composition precede the onset of inflammation. This is consistent with previous findings in IBD patients, where the difference in mucosal samples was even more pronounced.^49^

The early disease onset shortly after weaning appears to contrast with humans, in which the majority of early-onset IBD patients have an age of ~10 years at diagnosis.^50^ The ‘weaning reaction’ of the immune system in response to concomitant microbiota alterations is accompanied by an upregulation of TNF, which is thought to be a disease trigger in the *TNF*^*ΔARE*^ animal and probably contributes to the early disease onset in the swine model.^51^ As in mice the reasons why only about 50% of heterozygous porcine mutants show disease manifestations remain to be elucidated. In the mouse model, it has been reported that the abundance of an immunomodulatory commensal correlates with ileitis disease severity, although a cross-model and cross-species significance remains controversial.^52^

Regarding the *TNF* mRNA decay kinetics, the pig model shows some differences to the IBD mouse published by Kontoyiannis *et al*.,^53^ in which only the ARE has been deleted while in the swine model the ARE and CDE1 sequence was removed. We observed a comparable effect of LPS stimulus on the transcription of *TNF* in PBMC-derived macrophages, but a more pronounced increase in transcript half-life. This is in line with a recently published mouse model showing that disease severity increased with concurrent deletions of ARE and CDEs, where the authors suggested an impact of the mutations on post-transcriptional *TNF* mRNA regulation.^54^

Importantly, the *TNF*^*ΔARE*^ pigs show transmural inflammation not only in the terminal ileum as in *TNF*^*ΔARE*^ mice, but also a robust disease manifestation in the proximal colon as seen in humans. This is probably due to the fact that pigs, like humans, have their primary site for ingesta fermentation in the colon compared to caecal fermentation in mice. Since luminal microbes mediate intestinal fermentation, its anatomical localization directly affects the constituents of the gut microbiota and thus microbe–host interactions.^55^ Host-specific colonization of bacterial communities was also confirmed by the separation between microbiota profiles in the two models. On the other hand, clustering of the luminal and mucosal microbiota from *TNF*^*ΔARE/+*^ mice or a subset of human IBD patients with active disease and *TNF*^*ΔARE/ΔARE*^ pigs with strong inflammation suggests a species-independent composition of the microbiota correlating with inflammation severity.

In mice and humans, microbial composition is typically measured from faecal samples. It does not necessarily reflect bacterial diversity along the GI tract. In line with this, the cross-genotype comparison of bacterial microbiota alterations in stool provided only a weak reflection of the conditions observed for affected intestinal mucosal biopsies. The option to carry out repeated endoscopies or work with cannulated pigs will provide a better understanding of the local interactions between gut microbiota, inflammation, and nutritional or therapeutic interventions. None of this is possible in human patients. Like mice, pigs can be reared under GF conditions. Importantly, microbiome transfer from humans to pigs results in a gut microbiota closely resembling that of the human donor.^14^ Novel diagnostic technologies can be assessed at the human scale.^9^

In summary, this work reports on *TNF*^*ΔARE*^ pigs as a translational animal model for IBD. The *TNF*^*ΔARE*^ pigs recapitulate major characteristics of human CD, including ulcerative transmural ileocolitis, increased abundance of proinflammatory cytokines, impaired integrity of the intestinal epithelial cell barrier, immune cell infiltration and changes in dysbiotic microbial communities. This model enables human-scale and long-term studies to assess diagnostic, nutritional or microbial interventions. The pig model is not intended to substitute the mouse models, but rather to fill the gap for translating findings to the clinic. The value of pig models for digestive disease research has been frequently demonstrated,^9,56,57^ and the *TNF*^*ΔARE*^ pigs will undoubtedly become an important asset for patient-relevant translational CD studies.

## Supplementary Material

jjad034_suppl_Supplementary_Figure_S1

jjad034_suppl_Supplementary_Figure_S2

jjad034_suppl_Supplementary_Figure_Legends

## Data Availability

The accession number for the IBD patient cohort [HSCT-treated Crohn’s disease patients] raw 16S rRNA gene sequencing data reported in this paper was accessed via the Sequence Read Archive [SRA: PRJNA565903]. The accession number for the raw 16S rRNA gene sequencing data as well as metadata information of the pig and mouse models reported in this paper are available via the Sequence Read Archive [SRA: PRJNA907809]. Software programs used to analyse the data are either freely or commercially available. All other data relevant to the study are included in the article. Additional data are available on request.

## References

[1] Chang JT. Pathophysiology of inflammatory bowel diseases. N Engl J Med 2020;383:2652–64. 33382932 10.1056/NEJMra2002697

[2] Ng SC , Shi HY, Hamidi N, et al. Worldwide incidence and prevalence of inflammatory bowel disease in the 21st century: a systematic review of population-based studies. The Lancet 2017;390:2769–78. 10.1016/S0140-6736(17)32448-0 29050646

[3] Jostins L , Ripke S, Weersma RK, et al; International IBD Genetics Consortium (IIBDGC). Host–microbe interactions have shaped the genetic architecture of inflammatory bowel disease. Nature 2012;491:119–24. 23128233 10.1038/nature11582 PMC3491803

[4] Neurath MF. Animal models of inflammatory bowel diseases: illuminating the pathogenesis of colitis, ileitis and cancer. Dig Dis 2012;30:91–4. 10.1159/000341131 23075875

[5] Jergens AE , Sonea IM, O’Connor AM, et al. Intestinal cytokine mRNA expression in canine inflammatory bowel disease: a meta-analysis with critical appraisal. Comp Med 2009;59:153–62. 19389307 PMC2703145

[6] Gozalo A , Dagle GE, Montoya E, Weller RE. Spontaneous terminal ileitis resembling Crohn disease in captive tamarins. J Med Primatol 2002;31:142–6. 12190855 10.1034/j.1600-0684.2002.01002.x

[7] Taylor NP. *Synlogic scraps ammonia-lowering drug after early phase fail* , 2019. https://www.fiercebiotech.com/biotech/synlogic-scraps-ammonia-lowering-drug-after-early-phase-fail. Accessed March 7, 2022.

[8] Flisikowska T , Merkl C, Landmann M, et al. A porcine model of familial adenomatous polyposis. Gastroenterology 2012;143:1173–1175.e7. 22864254 10.1053/j.gastro.2012.07.110

[9] Rogalla S , Flisikowski K, Gorpas D, et al. Biodegradable fluorescent nanoparticles for endoscopic detection of colorectal carcinogenesis. Adv Funct Mater 2019;29:1904992. 33041743 10.1002/adfm.201904992 PMC7546531

[10] Ziegler A , Gonzalez L, Blikslager A. Large animal models: the key to translational discovery in digestive disease research. Cell Mol Gastroenterol Hepatol 2016;2:716–24. 28090566 10.1016/j.jcmgh.2016.09.003 PMC5235339

[11] Roura E , Koopmans S-J, Lallès J-P, et al. Critical review evaluating the pig as a model for human nutritional physiology. Nutr Res Rev 2016;29:60–90. 27176552 10.1017/S0954422416000020

[12] Wylensek D , Hitch TCA, Riedel T, et al. A collection of bacterial isolates from the pig intestine reveals functional and taxonomic diversity. Nat Commun 2020;11:6389. 33319778 10.1038/s41467-020-19929-w PMC7738495

[13] Forster SC , Kumar N, Anonye BO, et al. A human gut bacterial genome and culture collection for improved metagenomic analyses. Nat Biotechnol 2019;37:186–92. 30718869 10.1038/s41587-018-0009-7 PMC6785715

[14] Pang X , Hua X, Yang Q, et al. Inter-species transplantation of gut microbiota from human to pigs. ISME J 2007;1:156–62. 18043625 10.1038/ismej.2007.23

[15] Xiao L , Estellé J, Kiilerich P, et al. A reference gene catalogue of the pig gut microbiome. Nat Microbiol 2016;1:16161. 27643971 10.1038/nmicrobiol.2016.161

[16] Yang H , Wu J, Huang X, et al. ABOgenotype alters the gut microbiota by regulating GalNAc levels in pigs. Nature 2022;606:358–367. 10.1038/s41586-022-04769-z PMC9157047 35477154

[17] Concordet J-P , Haeussler M. CRISPOR: intuitive guide selection for CRISPR/Cas9 genome editing experiments and screens. Nucleic Acids Res 2018;46:W242–5. 29762716 10.1093/nar/gky354 PMC6030908

[18] Petersen B , Frenzel A, Lucas-Hahn A, et al. Efficient production of biallelic GGTA1 knockout pigs by cytoplasmic microinjection of CRISPR/Cas9 into zygotes. Xenotransplantation 2016;23:338–46. 27610605 10.1111/xen.12258

[19] Kurome M , Kessler B, Wuensch A, Nagashima H, Wolf E. Nuclear transfer and transgenesis in the pig. Methods Mol Biol 2015;1222:37–59. 25287337 10.1007/978-1-4939-1594-1_4

[20] Andersen CL , Jensen JL, Ørntoft TF. Normalization of real-time quantitative reverse transcription-PCR data: a model-based variance estimation approach to identify genes suited for normalization, applied to bladder and colon cancer data sets. Cancer Res 2004;64:5245–50. 15289330 10.1158/0008-5472.CAN-04-0496

[21] Pfaffl MW , Tichopad A, Prgomet C, Neuvians TP. Determination of stable housekeeping genes, differentially regulated target genes and sample integrity: BestKeeper--Excel-based tool using pair-wise correlations. Biotechnol Lett 2004;26:509–15. 15127793 10.1023/b:bile.0000019559.84305.47

[22] Khaloian S , Rath E, Hammoudi N, et al. Mitochondrial impairment drives intestinal stem cell transition into dysfunctional Paneth cells predicting Crohn’s disease recurrence. Gut 2020;69:1939–51. 32111634 10.1136/gutjnl-2019-319514 PMC7569388

[23] Metwaly A , Dunkel A, Waldschmitt N, et al. Integrated microbiota and metabolite profiles link Crohn’s disease to sulfur metabolism. Nat Commun 2020;11:4322. 32859898 10.1038/s41467-020-17956-1 PMC7456324

[24] Bazanella M , Maier TV, Clavel T, et al. Randomized controlled trial on the impact of early-life intervention with bifidobacteria on the healthy infant fecal microbiota and metabolome. Am J Clin Nutr 2017;106:1274–86. 28877893 10.3945/ajcn.117.157529

[25] Kozich JJ , Westcott SL, Baxter NT, Highlander SK, Schloss PD. Development of a dual-index sequencing strategy and curation pipeline for analyzing amplicon sequence data on the MiSeq Illumina sequencing platform. Appl Environ Microbiol 2013;79:5112–20. 23793624 10.1128/AEM.01043-13 PMC3753973

[26] Lagkouvardos I , Kläring K, Heinzmann SS, et al. Gut metabolites and bacterial community networks during a pilot intervention study with flaxseeds in healthy adult men. Mol Nutr Food Res 2015;59:1614–28. 25988339 10.1002/mnfr.201500125

[27] Lagkouvardos I , Joseph D, Kapfhammer M, et al. IMNGS: A comprehensive open resource of processed 16S rRNA microbial profiles for ecology and diversity studies. Sci Rep 2016;6:33721. 27659943 10.1038/srep33721 PMC5034312

[28] Edgar RC , Haas BJ, Clemente JC, Quince C, Knight R. UCHIME improves sensitivity and speed of chimera detection. Bioinformatics 2011;27:2194–200. 21700674 10.1093/bioinformatics/btr381 PMC3150044

[29] Lagkouvardos I , Fischer S, Kumar N, Clavel TR. A transparent and modular R pipeline for microbial profiling based on 16S rRNA gene amplicons. PeerJ 2017;5:e2836. 28097056 10.7717/peerj.2836 PMC5234437

[30] Quast C , Pruesse E, Yilmaz P, et al. The SILVA ribosomal RNA gene database project: improved data processing and web-based tools. Nucleic Acids Res 2013;41:D590-6. 23193283 10.1093/nar/gks1219 PMC3531112

[31] Kumar S , Stecher G, Li M, Knyaz C, Tamura K. MEGA X: molecular evolutionary genetics analysis across computing platforms. Mol Biol Evol 2018;35:1547–9. 29722887 10.1093/molbev/msy096 PMC5967553

[32] Barreau C , Paillard L, Osborne HB. AU-rich elements and associated factors: are there unifying principles? Nucleic Acids Res 2005;33:7138–50. 16391004 10.1093/nar/gki1012 PMC1325018

[33] Parameswaran N , Patial S. Tumor necrosis factor-α signaling in macrophages. Crit Rev Eukaryot Gene Expr 2010;20:87–103. 21133840 10.1615/critreveukargeneexpr.v20.i2.10 PMC3066460

[34] Danese S , Vermeire S, Hellstern P, et al. Randomised trial and open-label extension study of an anti-interleukin-6 antibody in Crohn’s disease (ANDANTE I and II). Gut 2019;68:40–8. 29247068 10.1136/gutjnl-2017-314562 PMC6839832

[35] Grimm MC , Elsbury SK, Pavli P, Doe WF. Interleukin 8: cells of origin in inflammatory bowel disease. Gut 1996;38:90–8. 8566866 10.1136/gut.38.1.90 PMC1382985

[36] Schaubeck M , Clavel T, Calasan J, et al. Dysbiotic gut microbiota causes transmissible Crohn’s disease-like ileitis independent of failure in antimicrobial defence. Gut 2016;65:225–37. 25887379 10.1136/gutjnl-2015-309333 PMC4752651

[37] Kurtz CB , Millet YA, Puurunen MK, et al. An engineered *E. coli* Nissle improves hyperammonemia and survival in mice and shows dose-dependent exposure in healthy humans. Sci Transl Med 2019;11. 10.1126/scitranslmed.aau7975 30651324

[38] Moser A , Luongo C, Gould K, McNeley M, Shoemaker A, Dove WA. A mouse model for intestinal and mammary tumorigenesis. Eur J Cancer 1995;31:1061–4. 10.1016/0959-8049(95)00181-h 7576992

[39] Lehr HA , Menger MD, Granger DN. Ischemia-reperfusion injury: enthusiasm in laboratory research but dilemma in clinical trials? Circulation 1994;90:1580. 8087970

[40] Rogers CS , Hao Y, Rokhlina T, et al. Production of CFTR-null and CFTR-DeltaF508 heterozygous pigs by adeno-associated ­virus-mediated gene targeting and somatic cell nuclear transfer. J Clin Invest 2008;118:1571–7. 18324337 10.1172/JCI34773 PMC2265103

[41] Guo C-H , Chen P-C, Yeh M-S, Hsiung D-Y, Wang C-L. Cu/Zn ratios are associated with nutritional status, oxidative stress, inflammation, and immune abnormalities in patients on peritoneal dialysis. Clin Biochem 2011;44:275–80. 21223959 10.1016/j.clinbiochem.2010.12.017

[42] Cherfane CE , Gessel L, Cirillo D, Zimmerman MB, Polyak S. Monocytosis and a low lymphocyte to monocyte ratio are effective biomarkers of ulcerative colitis disease activity. Inflamm Bowel Dis 2015;21:1769–75. 25993688 10.1097/MIB.0000000000000427 PMC5193095

[43] Felber S , Rosenthal P, Henton D. The BUN/creatinine ratio in localizing gastrointestinal bleeding in pediatric patients. J Pediatr Gastroenterol Nutr 1988;7:685–7. 3263488 10.1097/00005176-198809000-00011

[44] Lloyd-Price J , Arze C, Ananthakrishnan AN, et al; IBDMDB Investigators. Multi-omics of the gut microbial ecosystem in inflammatory bowel diseases. Nature 2019;569:655–62. 31142855 10.1038/s41586-019-1237-9 PMC6650278

[45] Albenberg L , Esipova TV, Judge CP, et al. Correlation between intraluminal oxygen gradient and radial partitioning of intestinal microbiota. Gastroenterology 2014;147:1055–63.e8. 25046162 10.1053/j.gastro.2014.07.020 PMC4252572

[46] Rigottier-Gois L. Dysbiosis in inflammatory bowel diseases: the oxygen hypothesis. ISME J 2013;7:1256–61. 23677008 10.1038/ismej.2013.80 PMC3695303

[47] Hutchinson E. Risk of IBD increases after *Salmonella* or *Campylobacter* gastroenteritis. Nat Rev Gastroenterol Hepatol 2009;6:561561.–561.

[48] Shao J , Li Z, Gao Y, et al. Construction of a ‘bacteria-metabolites’ co-expression network to clarify the anti-ulcerative colitis effect of flavonoids of *Sophora flavescens* Aiton by regulating the ‘host-microbe’ interaction. Front Pharmacol 2021;12:710052. 34721011 10.3389/fphar.2021.710052 PMC8553221

[49] Glymenaki M , Singh G, Brass A, et al. Compositional changes in the gut mucus microbiota precede the onset of colitis-induced inflammation. Inflamm Bowel Dis 2017;23:912–22. 28498157 10.1097/MIB.0000000000001118

[50] Benchimol EI , Mack DR, Nguyen GC, Snapper SB, Li W, Mojaverian N, Quach P, Muise AM. Incidence, outcomes, and health services burden of very early onset inflammatory bowel disease. Gastroenterology 2014;147:803–813.e7; quiz e14.e7; quiz e14-5. 24951840 10.1053/j.gastro.2014.06.023

[51] Guo F , Cai D, Li Y, et al. How early-life gut microbiota alteration sets trajectories for health and inflammatory bowel disease? Front Nutr 2021;8:690073. 34422881 10.3389/fnut.2021.690073 PMC8377158

[52] Metwaly A , Jovic J, Waldschmitt N, et al. Diet prevents the expansion of segmented filamentous bacteria and ileo-colonic inflammation in a model of Crohn’s disease. *bioRxiv *2022:2022.07.06.498810. doi:10.1101/2022.07.06.498810. 10.1186/s40168-023-01508-y PMC10064692 37004103

[53] Kontoyiannis D , Pasparakis M, Pizarro TT, Cominelli F, Kollias G. Impaired On/off regulation of TNF biosynthesis in mice lacking TNF AU-rich elements. Immunity 1999;10:387–98. 10204494 10.1016/s1074-7613(00)80038-2

[54] Clayer E , Dalseno D, Kueh A, et al. Severe impairment of TNF post-transcriptional regulation leads to embryonic death. iScience 2020;23:101726101726. 10.1016/j.isci.2020.101726 PMC7658709 33210082

[55] Rose EC , Blikslager AT, Ziegler AL. Porcine models of the intestinal microbiota: the translational key to understanding how gut commensals contribute to gastrointestinal disease. Front Vet Sci 2022;9:834598. 35400098 10.3389/fvets.2022.834598 PMC8990160

[56] Troya J , Krenzer A, Flisikowski K, et al. New concept for colonoscopy including side optics and artificial intelligence. Gastrointest Endosc 2022;95:794–8. 34929183 10.1016/j.gie.2021.12.003

[57] Yim JJ , Harmsen S, Flisikowski K, et al. A protease-activated, near-infrared fluorescent probe for early endoscopic detection of premalignant gastrointestinal lesions. Proc Natl Acad Sci U S A 2021;118. 10.1073/pnas.2008072118 PMC7817203 33443161

