# Supplementary figures and images for: TNF ΔARE Pigs: A Translational Crohn’s Disease Model
Source: J Crohns Colitis. 2023 Feb 23;17(7):1128–38. doi: 10.1093/ecco-jcc/jjad034 (PMC10320488; doi:10.1093/ecco-jcc/jjad034)

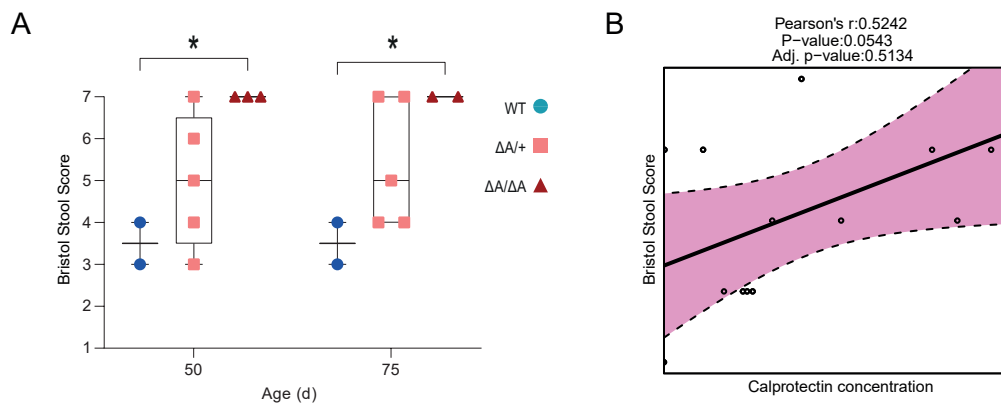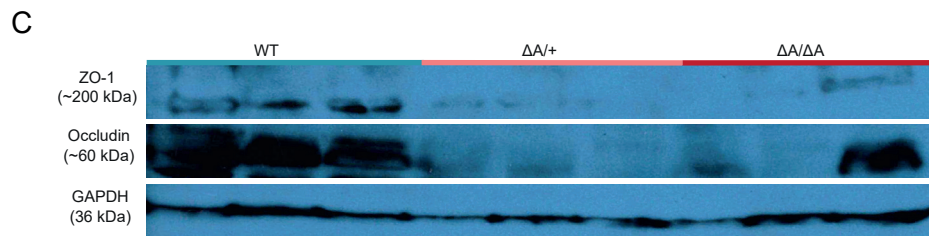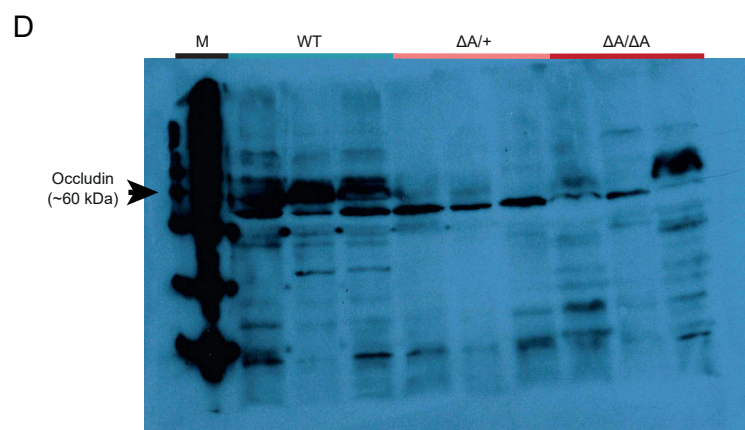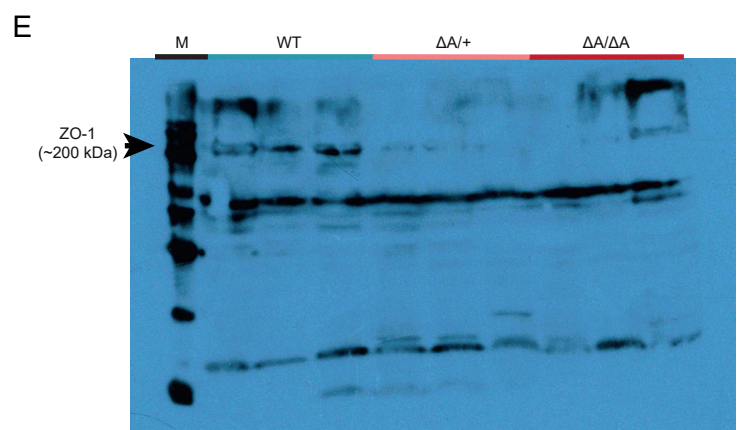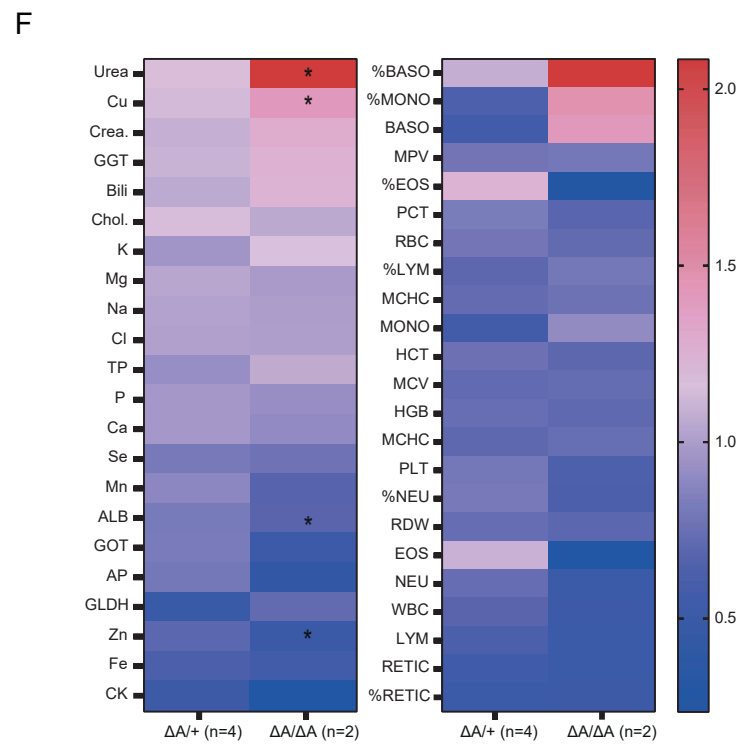

Supplement: jjad034_suppl_Supplementary_Figure_S1 [file jjad034_suppl_supplementary_figure_s1.pdf]
