# Supplementary material for: TNF ΔARE Pigs: A Translational Crohn’s Disease Model
Source: J Crohns Colitis. 2023 Feb 23;17(7):1128–38. doi: 10.1093/ecco-jcc/jjad034 (PMC10320488; doi:10.1093/ecco-jcc/jjad034)
Supplement: jjad034_suppl_Supplementary_Figure_S2 [file jjad034_suppl_supplementary_figure_s2.pdf]

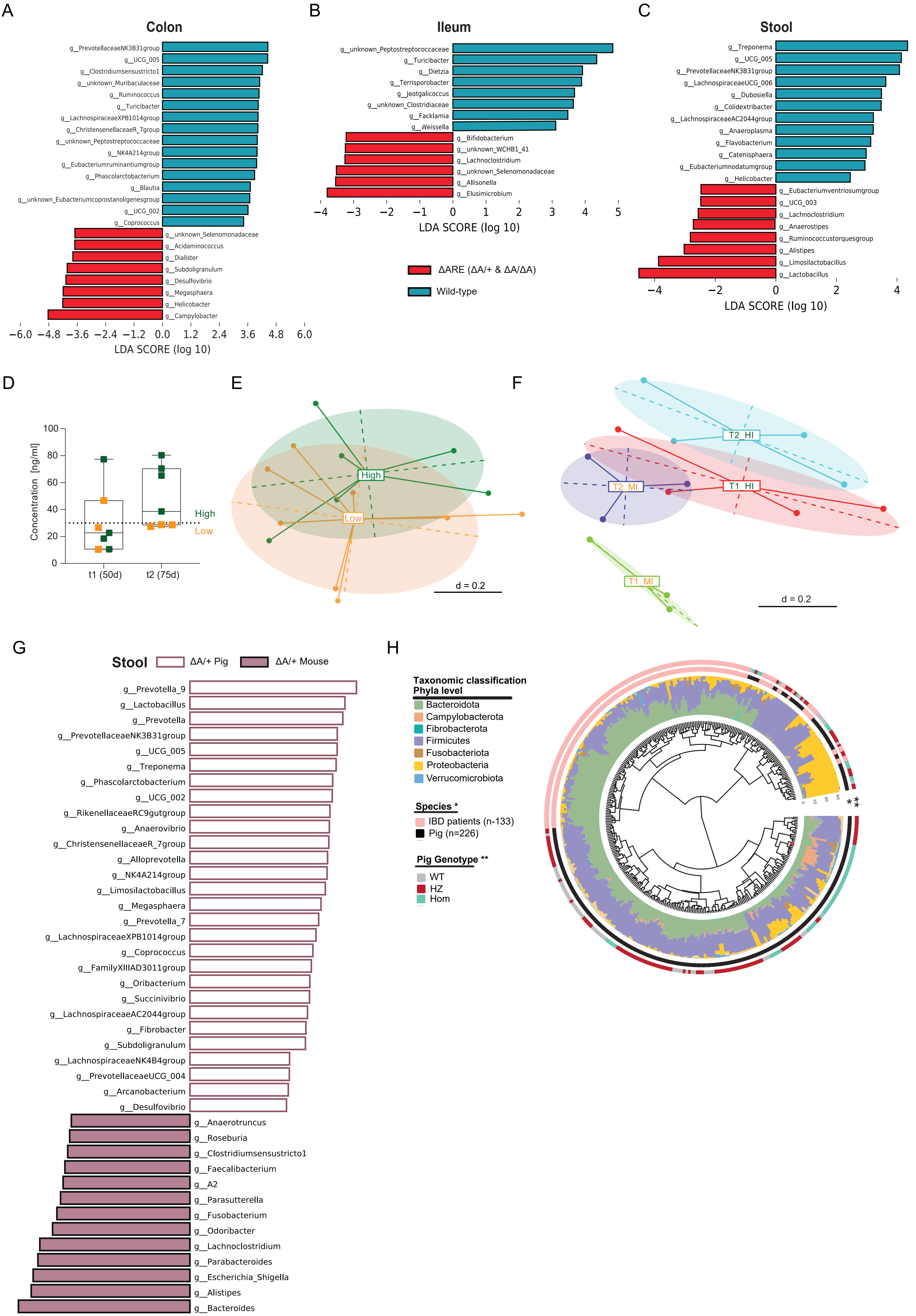
A
Colon
g__PrevotellaceaeNK3B31group
g__UCG_005
g__Clostridiumsensustricto1
g__unknown_Muribaculaceae
g__Ruminococcus
g__Turicibacter
g__LachnospiraceaeXPB1014group
g__ChristensenellaceaeR_7group
g__unknown_Peptostreptococcaceae
g__NK4A214group
g__Eubacteriumruminantiumgroup
g__Phascolarctobacterium
g__Blautia
g__unknown_Eubacteriumcoprostanoligenesgroup
g__UCG_002
g__Coprococcus
g__unknown_Selenomonadaceae
g__Acidaminococcus
g__Dialister
g__Subdoligranulum
g__Desulfovibrio
g__Megasphaera
g__Helicobacter
g__Campylobacter
−6.0 −4.8 −3.6 −2.4 −1.2 0.0 1.2 2.4 3.6 4.8 6.0
LDA SCORE (log 10)
B
Ileum
g__unknown_Peptostreptococcaceae
g__Turicibacter
g__Dietzia
g__Terrisporobacter
g__Jeotgalicoccus
g__unknown_Clostridiaceae
g__Facklamia
g__Weissella
g__Bifidobacterium
g__unknown_WCHB1_41
g__Lachnoclostridium
g__unknown_Selenomonadaceae
g__Allisonella
g__Elusimicrobium
−4 −3 −2 −1 0 1 2 3 4 5
LDA SCORE (log 10)
ΔARE (ΔA/+ & ΔA/ΔA)
Wild-type
C
Stool
g__Treponema
g__UCG_005
g__PrevotellaceaeNK3B31group
g__LachnospiraceaeUCG_006
g__Dubosiella
g__Colidextribacter
g__LachnospiraceaeAC2044group
g__Anaeroplasma
g__Flavobacterium
g__Catenisphaera
g__Eubacteriumnodatumgroup
g__Helicobacter
g__Eubacteriumventriosumgroup
g__UCG_003
g__Lachnoclostridium
g__Anaerostipes
g__Ruminococcustorquesgroup
g__Alistipes
g__Limosilactobacillus
g__Lactobacillus
−4 −2 0 2 4
LDA SCORE (log 10)
D
Concentration [ng/ml]
100
80
60
40
20
0
High
Low
t1 (50d)
t2 (75d)
E
High
Low
d = 0.2
F
T2_HI
T2_MI
T1_HI
T1_MI
d = 0.2
G
Stool
ΔA/+ Pig
ΔA/+ Mouse
g__Prevotella_9
g__Lactobacillus
g__Prevotella
g__PrevotellaceaeNK3B31group
g__UCG_005
g__Treponema
g__Phascolarctobacterium
g__UCG_002
g__RikenellaceaeRC9gutgroup
g__Anaerovibrio
g__ChristensenellaceaeR_7group
g__Alloprevotella
g__NK4A214group
g__Limosilactobacillus
g__Megasphaera
g__Prevotella_7
g__LachnospiraceaeXPB1014group
g__Coprococcus
g__FamilyXIIIAD3011group
g__Oribacterium
g__Succinivibrio
g__LachnospiraceaeAC2044group
g__Fibrobacter
g__Subdoligranulum
g__LachnospiraceaeNK4B4group
g__PrevotellaceaeUCG_004
g__Arcanobacterium
g__Desulfovibrio
g__Anaerotruncus
g__Roseburia
g__Clostridiumsensustricto1
g__Faecalibacterium
g__A2
g__Parasutterella
g__Fusobacterium
g__Odoribacter
g__Lachnoclostridium
g__Parabacteroides
g__Escherichia_Shigella
g__Alistipes
g__Bacteroides
H
Taxonomic classification
Phyla level
Bacteroidota
Campylobacterota
Fibrobacterota
Firmicutes
Fusobacteriota
Proteobacteria
Verrucomicrobiota
Species *
IBD patients (n-133)
Pig (n=226)
Pig Genotype **
WT
HZ
Hom
*
**
