# Supplementary material for: TNF ΔARE Pigs: A Translational Crohn’s Disease Model
Source: J Crohns Colitis. 2023 Feb 23;17(7):1128–38. doi: 10.1093/ecco-jcc/jjad034 (PMC10320488; doi:10.1093/ecco-jcc/jjad034)
Supplement: jjad034_suppl_Supplementary_Figure_Legends [file jjad034_suppl_supplementary_figure_legends.docx]

**Supplementary Figure 1**

*(A) Faecal Bristol stool scores for two post-weaning time points t1 (50 days) and t2 (75 days) derived from wild-type (n=2), TNF^ΔARE/+^ (n=5) and TNF^ΔARE/ΔARE^ (n=3 and 2) pigs of the same litter. A Bristol stool score of 1 indicates hard lumpy stool consistency, while a score of 7 indicates watery, entirely liquid stool. (B) Pearson correlation coefficient of bristol stool scores and Calprotectin concentrations of respective samples. Shown are individual sample-specific values, lower and upper boundaries of the predicted interval, as determined using the R function predict, and the linear fitted line. (C) Selected sections and GAPDH protein expression in colonic tissue biopsies from 3 wild-type, 3 TNF^ΔARE/+^ and 3 TNF^ΔARE/ΔARE^ pigs. M, size marker. (E, F) Respective complete Western blot images for ZO-1 and Occludin. Band locations and sizes of ZO-1 and Occludin are indicated. (F) Heatmap of the complete blood count of TNF^ΔARE/+^ and TNF^ΔARE/ΔARE^ pigs compared to wild-type littermates (n=3). *, significant differences. (GOT: glutamate-oxalacetate-transaminase; GLDH: glutamate-dehydrogenase; GGT: y-Glutamyl-Transferase; Billi: Bilirubin; AP: alkaline phosphatase; Chol.: cholesterol; TP: total protein; ALB: albumin; RBC: red blood cells; HCT: haematocrit; HGB: haemoglobin; MCV: mean corpuscular volume; MCHC: Mean corpuscular haemoglobin concentration; RDW: red cell distribution; RETIC: reticulocyte; WBC: white blood cell; NEU: neutrophils; LYM: lymphocytes; MONO: monocytes; EOS: eosinophils; BASO: basophils; PLT: platelets; MPV: mean platelet volume; PCT: procalcitonin).*

**Supplementary Figure 2**

*(A, B, C) Linear discriminant analysis effect size (LEfSe) indicating differentially enriched bacterial groups at the genus level of ileal, colonic and stool microbiota based on genotype. Red: TNF^ΔARE^ and Blue: wild-type. (D) Faecal Calprotectin concentrations measured by ELISA in stool samples (n=7) for two consecutive post-weaning time points, t1 (50 days) and t2 (75 days). A threshold at 30 ng/ml separates highly inflamed from mildly inflamed samples. Green color indicates high Calprotectin concentration at t2, while orange color indicates low Calprotectin concentration at t2. (E) MDS plot of microbial profiles of respective faecal samples stratified by a high or low Calprotectin concentration, respectively. (F) MDS plot of microbial profiles of respective faecal samples stratified by a high (“HI”) or low Calprotectin concentration at t2 (“MI”), respectively. (G) LEfSe indicating differentially enriched bacterial groups at the genus level of faecal microbiota based on species. Empty: TNF^ΔARE^ mouse and Filled: TNF^ΔARE^ pig. (H) Phylogenetic tree showing the similarities between microbiota profiles based on generalized UniFrac distances in luminal and mucosal microbiota derived from wild-type and TNF^ΔARE^ pigs and human IBD patients. Individual taxonomic composition at the phylum level is shown as stacked bar plots around the phylogram. Innermost ring shows stratification based on species; human or pig and the outer ring shows stratification based on genotype, wild-type (grey); TNF^ΔARE/+^ (red) and TNF^ΔARE/ΔARE^ (green).*
